# What of Cataphoresis?—Is It Practicable?—Is It Desirable?

**Published:** 1897-04

**Authors:** 


### EDITORIAL.

### What of Cataphoresis?—Is it Practicable?—Is it Desirable?

These questions are asked almost daily. While we will not here attempt to answer these queries in detail, two or three things may be said. And first, cataphoresis for the treatment of sensitive dentine or exposed tooth-pulps will not be successful in the hands of those ignorant of the properties of the very subtle agent used in this work; a very small percent of the profession, indeed, have given any attention or study to the subject of electricity, and are wholly incompetent to handle it with any definite results, and ought not to attempt its employment in the absence of a reasonable knowledge about it. The knowledge here indicated implies, of course, an acquaintance of the various instruments and appliances with which the agent is manipulated.

To the second inquiry it may be replied, yes—in the hands of those competent to use it. From this it must not be inferred that the desired results can be obtained alike in all cases; there is an infinite variety in the susceptibility of different cases. This is true in regard to all methods of treatment, and of the action of all medical agents. That in the large proportion of cases of sensitive dentine an entire reduction of that condition can be effected there is no question; but that in the hands of the most skillful there will be occasional failures is equally certain. In many cases in which pulps are to be removed it serves an admirable purpose. In several cases in which we have used this method for anæsthetising the dental pulp for removal, it was in each an absolute success, but we do not accept that as prophetic of like results in all other cases.

To the third query—Is it desirable?—Yes, so far as it will accomplish the object sought. Anything is desirable that will allay acute sensitiveness of dentine when it is to be operated upon, and that will not be attended with disastrous results. Cataphoresis seems, in the hands of the intelligent and skillful, to be quite as effective as any other means hitherto used, and with less objectionable after-results than some other agents. In the removal of pulps by the use of cataphoresis, employing a due amount of care and skill, the liability to ill results is reduced to the minimum.

